# Effects of Breastfeeding on Stress Measured by Saliva Cortisol Level and Perceived Stress

**DOI:** 10.31372/20200503.1100

**Published:** 2020

**Authors:** Kiyoko Mizuhata, Hatsumi Taniguchi, Mieko Shimada, Naoko Hikita, Seiichi Morokuma

**Affiliations:** a Dokkyo Medical University, Japan; b Kyushu University, Japan; c Osaka University, Japan

**Keywords:** breastfeeding, cortisol, postpartum depression, psychological stress

## Abstract

**Purpose:** The effects of breastfeeding on postpartum depression symptoms and stress using physiological measures require investigation.

**Background:** Breastfeeding suppresses the secretion of cortisol. Oxytocin levels correlate negatively with symptoms of postpartum depression.

**Aim:** To investigate the effects of breastfeeding on stress and postpartum depression.

**Methods:** We examined 79 breastfeeding women using the Edinburgh Postnatal Depression Scale, the Perceived Stress Scale-10, and the Breastfeeding Self-Efficacy Scale, and measured the salivary cortisol levels before and after breastfeeding.

**Findings:** There was a negative correlation between the duration of suckling and changes in salivary cortisol levels following breastfeeding (*r*_s_ = −0.333, *p* < 0.05). Salivary cortisol levels immediately following breastfeeding were significantly lower compared to mothers who used mixed feeding methods (*p* < 0.001). Breastfeeding mothers had lower perceived stress than mothers using mixed feeding methods (β = −0.260, *p* < 0.05). There was no association between breastfeeding and postpartum depression; however, there was an association between postpartum depression and perceived stress (*β* = 0.526, *p* < 0.001).

**Conclusion:** Salivary cortisol levels significantly decreased following breastfeeding, with longer suckling times correlating with lower cortisol levels. Breastfeeding reduced stress and increased breastfeeding self-efficacy.

## Introduction

Breastfeeding affects infant health, child’s long-term health, and maternal health (Chowdhury et al., 2015; Sankar et al., 2015). However, the evidence on the association between breastfeeding and postpartum depression is unclear (Dennis & McQueen, 2009).

Nipple stimulation during breastfeeding triggers the release of oxytocin, a neuropeptide hormone (Heinrichs et al., 2001). Research on the effects of breastfeeding in rats has revealed evidence that supports a breastfeeding-induced “stress-buffering” hypothesis. Animal research indicates that the hypothalamic–pituitary axis responses to stress are reduced during breastfeeding (Higuchi, Neguro, & Arita, 1989; Lightman, 1992). In humans, breastfeeding is associated with a suppressed hypothalamic–pituitary axis response to exercise stress (Altemus, Deuster, Galliven, Carter, & Gold, 1995). Breastfeeding an infant for 15 min decreases state anxiety (Heinrichs et al., 2001). The available data suggest that breastfeeding suppresses maternal stress.

Oxytocin levels during pregnancy are negatively correlated with symptoms of postpartum depression (Skrundz, Bolten, Nast, Hellhammer, & Meinlschmidt, 2011). Postpartum depression is an obstacle to breastfeeding (Galler, Harrison, Biggs, Ramsey, & Forde, 1999; Kehler, Chaput, & Tough, 2009). The symptoms of anxiety and depression during pregnancy tend to worsen approximately six months after childbirth if the mother discontinues breastfeeding (Ystrom, 2012), and exclusive breastfeeding may be useful in reducing symptoms of depression that develop during the first three months after delivery (Figueredo, Mattar, & Abrão, 2013). These results suggest that breastfeeding may reduce the risk of stress or postpartum depression. Therefore, investigating whether breastfeeding has the potential to reduce the risk of stress and postpartum depression is crucial.

This study aimed to determine whether suckling stimulation inhibits stress and the effects of breastfeeding on perceived stress and postpartum depression.

## Methods

### Study Participants

This is a cross-sectional study. We recruited breastfeeding mothers in postpartum days 14 to 120 at their two-week postpartum medical checkup or one-month checkup or at a postpartum mother-and-child class at a primary medical facility in Japan. The inclusion criteria were mothers of a singleton birth, infants without serious disease, no history of maternal mental illness, and Japanese literacy. The survey period was 6 months, from August 2019 to January 2020.

### Data Collection Methods

Participants’ demographic data, medical records, and saliva were collected by a researcher. An anonymous, self-reported questionnaire to be completed during the 10- to 15-min waiting period was distributed during their visit. Immediately before and after breastfeeding, in both exclusively breastfeeding and mixed-feeding mothers, 1.5 mL of saliva was collected using the passive drool method and stored at −80 °C until analysis. Investigators recorded the start and end times of breastfeeding. Information on pregnancy and delivery was obtained from the participants’ medical records.

### Salivary Cortisol Measurement

Salivary cortisol levels were used as an objective measurement of acute stress response. Saliva was collected twice just before and immediately after breastfeeding in 72 of 79 participants. Cortisol was assayed from saliva specimens using a competitive enzyme immunoassay kit from the Yanaihara Institute Inc. (Shizuoka, Japan). The measurement was performed by the Yanaihara Institute Inc., Shizuoka Prefecture, Japan. Coefficients of variation from duplicate measurements < 15% were used in the analyses although Calvi et al. encourages researchers to maintain high standards for intra-assay coefficients of variation (<10%) (Calvi et al., 2017).

The difference in salivary cortisol levels, measured in μg/mL, just before and immediately after breastfeeding, divided by the level just before breastfeeding was recorded as the proportional change (%) for each individual.

### Survey Items

The following demographic characteristics were collected: age, parity, number of days following delivery at the time of the survey, gestational age at delivery, mode of delivery, birth weight, infant’s sex, marital status, employment status (plans to return to work, and timing of return), partner’s employment status, educational background, support and living conditions following delivery, and sleep time.

### Postpartum Depression

The postpartum depression symptoms were evaluated by assessing the potential factors affecting bonding using the Japanese version of the Edinburgh Postnatal Depression Scale (EPDS, Okano et al., 1996) developed by Cox, Holden, and Sagovsky (1987). The scale is a 10-item self-rating questionnaire. Responses are scored on a four-point Likert scale, with 0–3 points per item and a total possible score of 30 points. We adopted a threshold score ≥ 9 points in detecting the probable symptoms of depression for postpartum Japanese women, as suggested by Yamashita, Yoshida, Nakano, and Tashiro (2000). In this study, the Cronbach alpha coefficient for the EPDS was 0.859.

### Perceived Stress

Perceived stress was evaluated using the Japanese version of the Perceived Stress Scale-10 (PSS-10), developed by Cohen, Kamarck, and Mermelstein (1983). The Japanese version was developed by Sumi (2006), and its reliability and validity have been confirmed. Responses are scored on a five-point Likert scale, with 0–4 points per item and a total possible score of 40 points, with a higher score indicating higher levels of perceived stress. In this study, the Cronbach alpha coefficient for PSS-10 was 0.746.

### Breastfeeding

We obtained information on the presence or absence of sleep deprivation and fatigue due to breastfeeding, breastfeeding method (exclusive breastfeeding, mixed feeding with predominant breastfeeding, or mixed feeding with predominant formula feeding), frequency of breastfeeding per day, duration of suckling per feed, number of supplemental formula feeds per day, presence or absence of difficulties in breastfeeding, type of difficulties, and self-efficacy for breastfeeding.

Self-efficacy, a personal judgment on how well one can execute a course of action, was measured using the Japanese version of the 14-item self-reporting Breastfeeding Self-Efficacy Scale (BSES) developed by Dennis (2003). The Japanese version was developed by Otsuka, Dennis, Tatsuoka, and Jimba (2008), and its reliability and validity have been confirmed. Responses are scored on a five-point Likert scale, with 0–5 points per item and maximum possible total score of 70 points. A higher score indicated greater self-efficacy. In this study, the Cronbach alpha coefficient of BSES was 0.947.

### Data Analysis

SPSS Statistics version 25.0 for Windows (IBM Corp., Armonk, NY, USA) was used in the statistical analyses, with a two-sided significance level set at <0.05. The chi-square test was used to determine significance of categorical data. In the event of non-normal distribution, the Kruskal–Wallis test was used to compare three-group means, the Mann–Whitney *U* test to compare the mean of two groups, and the Wilcoxon signed-rank test to determine differences between two groups. Multiple regression analyses were performed to explore possible associations between EPDS/PSS-10 scores and breastfeeding.

### Ethical Considerations

The purpose and methods of the study were explained to the participants. It was emphasized that participation was voluntary, with no adverse consequences for declining participation. Written informed consent was obtained after discussing how the data collected from the study would be managed and how confidentiality would be maintained. This study was conducted with the approval of the ethics review committee of the Osaka university hospital (approval number: 15539-5).

## Results

### Subject Attributes

Informed consent was obtained from 79 participants who met the eligibility criteria. Table 1 shows the participants’ demographic and postpartum characteristics. The average age of the participants was 32.3 ± 4.4 years. The average age of primiparous women was 30.9 years. There were 35 (44.3%) women who practiced exclusive breastfeeding and 44 (55.7%) who practiced mixed feeding. The mean postpartum days was 46.1, and the range was 14–112 days.

**Table 1:** Participants’ Demographic Characteristics (n = 79)

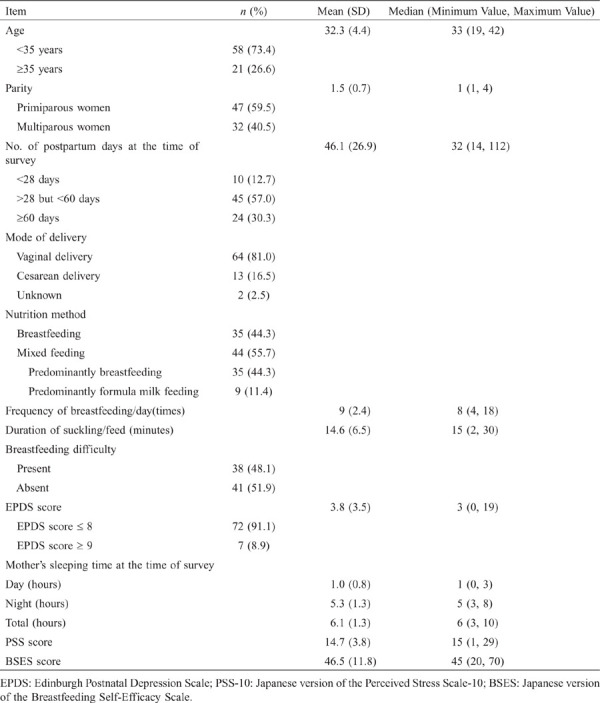

The mean EPDS score was 3.8 in a non-normal distribution, and seven women (8.9%) had an EPDS score ≥ 9, indicating symptoms for postpartum depression. The EPDS scores (median, 5; interquartile range, 1–7) of 38 mothers with breastfeeding difficulties were significantly higher than those of 41 mothers who had no breastfeeding difficulty (median, 2; interquartile range, 1–5; Mann–Whitney *U* test, *p* < 0.05).

The mean PSS-10 score was 14.7 in a non-normal distribution. The PSS-10 scores for mothers practicing mixed feeding (median, 15; interquartile range, 14–17) were higher than for mothers who were exclusively breastfeeding (median, 14; interquartile range, 11–14; Mann–Whitney *U* test: *p* < 0.01). Although there is a statistical difference between those who were breastfeeding and those who did not, the difference score of 1 is not clinically significant.

### Relationship between Postpartum Depression Symptoms and Breastfeeding

There were 33 (94.3%) mothers exclusively breastfeeding and 39 (88.6%) mothers who were practicing mixed feeding with EPDS score < 9. There was no significant association between feeding method and EPDS score (Mann–Whitney *U* test, *p* = 0.166). Mothers with postpartum depression symptoms had higher PSS-10 scores (median, 20; interquartile range, 18–23) than those without (median, 15; interquartile range, 13–16: Mann–Whitney *U* test, *p* < 0.01). The EPDS scores were negatively correlated with age (*r*_s_ = −0.300, *p* < 0.01) but were positively correlated with PSS-10 scores (*r*_s_ = 0.448, *p* < 0.001).

Table 2 shows the results of the multiple regression analyses that were performed using the EPDS score as the dependent variable. On single regression analysis, a significant correlation was noted between EPDS score and age, parity, delivery mode, and PSS-10 score. We set EPDS score as the dependent variable and age, parity, delivery mode, PSS-10 score, feeding method, BSES score, presence or absence of breastfeeding difficulty, frequency of breastfeeding per day, and sleep duration as independent variables on stepwise forced entry of multiple regression analysis.

**Table 2:** Multiple Regression Analysis using Edinburgh Postnatal Depression Scale (EPDS) as Dependent Variable (n = 79)

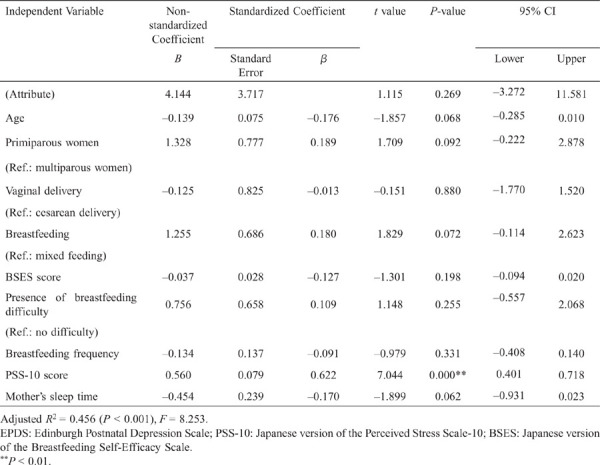

EPDS scores were significantly related to PSS-10 scores (*β* = 0.622, *p* < 0.001). However, there was no significant association between EPDS score and feeding method or any other variable. The adjusted *R*^2^ of the multiple regression model was 0.456 (*p* < 0.001).

### Relationship Between Perceived Stress and Breastfeeding

The PSS-10 scores were not significantly related to the number of days following delivery, maternal age, or parity. Table 3 shows the results of the multiple regression analyses that were performed using PSS-10 score as the dependent variable. On single regression analysis, a significant correlation was noted between PSS-10 and EPDS scores. We set the PSS-10 score as the dependent variable, and age, parity, EPDS score, feeding method, BSES score, presence or absence of breastfeeding difficulty, frequency of breastfeeding per day, and sleep duration attended as independent variables on stepwise forced entry of multiple regression analysis.

**Table 3:** Multiple Regression Analysis using Perceived Stress Scale-10 (PSS-10) as Dependent Variable (n = 79)

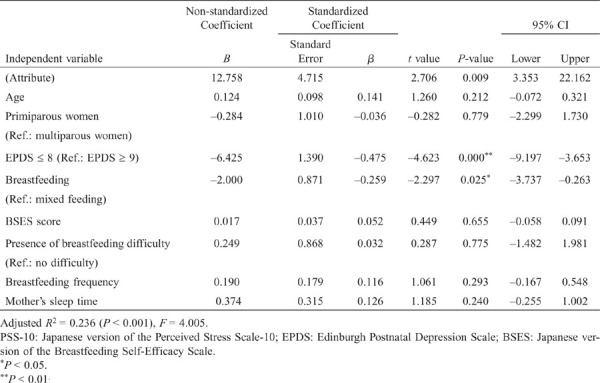

PSS-10 scores were significantly negatively weakly related to breastfeeding (*β* = −0.259, *p* < 0.05) and EPDS score ≤ 8 points (*β* = −0.475, *p* < 0.001). When multicollinearity was examined, the variance inflation factor value was < 2 in all variables, indicating absence of multicollinearity. The adjusted *R*^2^ of the multiple regression model was 0.236 (*p* < 0.001).

### Salivary Cortisol Levels and Relationship with Breastfeeding

Saliva was collected twice just before and immediately after breastfeeding in 72 of 79 participants. Coefficients of variation from duplicate measurements <15% were used in the analyses in 50 of 72 participants. Data of 20 of the 72 participants were excluded because of variation > 15%.

**Figure 1.**
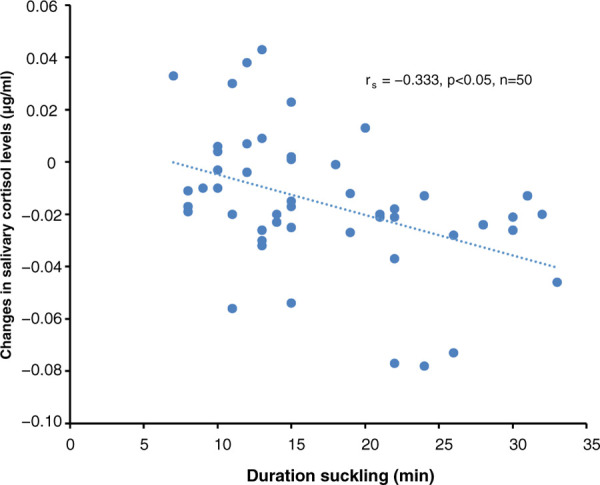
Suckling duration and changes in salivary cortisol level before and after breastfeeding. The mean suckling duration was 16.5 ± 7.0 min, and a significant negative correlation (*r*_s_ = −0.333 *p* < 0.05) was observed between the suckling duration and changes in pre- and post-lactation salivary cortisol levels.

The mean suckling duration recorded at the start and end times of breastfeeding by investigator was 16.5 ± 7.0 min. The sampling time lag just before and immediately after breastfeeding was < 35 min. A significant negative weak correlation (*r*_s_ = −0.333, *p* < 0.05) was observed between the suckling duration and changes in salivary cortisol levels (Figure 1).

The values, amount of change in the values, and rate of change in salivary cortisol level from before to after feeding are shown in Table 4. Salivary cortisol levels significantly decreased following breastfeeding (*n* = 50, Wilcoxon signed-rank test, *p* < 0.001), which was not clinically significant. The median change in salivary cortisol levels before and after breastfeeding was −0.011 g/dL.

**Table 4:** Changes in Salivary Cortisol Levels Before and After Breastfeeding and Rate of Change

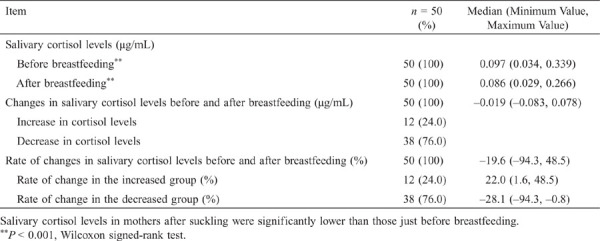

The median value of difference in salivary cortisol level just before and immediately after breastfeeding were 0.005 μg/dL (*n* = 22) in the exclusively breastfeeding group, median 0.021 μg/dL (*n* = 22) in the predominantly breastfeeding group, and median 0.017 μg/dL (n = 6) in the predominantly formula mixed feeding group, respectively (Table 5). In the mixed feeding group of the predominantly breastfeeding, salivary cortisol levels significantly decreased immediately after breastfeeding than just before (*n* = 22, pre-median 0.107 [0.068–0.148] μg/dL, post-median 0.086 [0.059–0.131] μg/dL) (Wilcoxon signed-rank test, *p* < 0.01).

**Table 5:** Salivary Cortisol Levels Before and After Breastfeeding for Each Nutrition Method (n = 50)

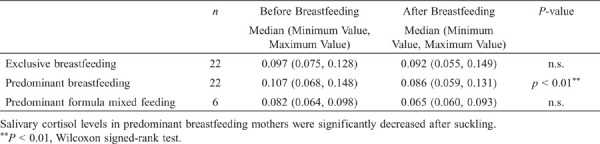

The mean suckling durations were 13.6 ± 6.6 min (n = 22) in the exclusively breastfeeding group, 16.4 ± 6.0 min (n = 22) in the predominantly breastfeeding group, and 11.8 ± 6.4 min (n = 6) in the predominantly formula mixed feeding group.

## Discussion

This study is the first to reveal stress reduction after breastfeeding measured through salivary cortisol levels in postpartum Japanese women. The relationship between breastfeeding and perceived stress was also revealed, and exclusively breastfeeding mothers were found to feel less stressed than mothers who used mixed feeding methods.

### Relationship between Duration of Suckling Stimulation and Saliva Cortisol Levels

We found a negative correlation between the suckling duration and changes in salivary cortisol levels before and after breastfeeding. This correlation indicated that the longer the suckling time, the lower the stress response.

Amico, Johnston, and Vagnucci (1994) studied six breastfeeding mothers and found that serum cortisol levels were considerably reduced after breastfeeding for 15 min. In a pilot study conducted by Neu, Pan, Haight, Fehringer, and Maluf (2017), salivary cortisol levels decreased in 20 mothers after breastfeeding. These studies were reinforced by our study in 50 mothers: the longer the suckling time, the lower the cortisol levels after breastfeeding.

Chiodera et al. (1991) measured the serum oxytocin and adrenocorticotropin hormone levels and reported that oxytocin levels were inversely correlated with hypothalamic-pituitary axis activity and cortisol secretion. Therefore, maternal stimulation by suckling promotes oxytocin secretion, resulting in suppression of hypothalamic-pituitary axis activity and reduction in cortisol in the mother during breastfeeding.

In this study, the salivary cortisol levels of 50 individuals just before and immediately after breastfeeding showed a significant decrease following breastfeeding. This decrease occurred to the greatest degree in the mixed feeding group of predominantly breastfeeding mothers. This study showed that the longer the suckling duration recorded at the start and end times of breastfeeding, the lower the salivary cortisol levels before and after breastfeeding. Therefore, the greatest decrease in cortisol level was observed in the group of predominantly breastfeeding mothers because they had the longest suckling duration in this study.

This finding suggests that, by continuing to nurse, the secretion of cortisol was suppressed even in mothers practicing mixed feeding and may have contributed to stress reduction.

Hammerfald, Eberle, Grau, Kinsperger, and Zimmermann (2006) reported that the maximum salivary cortisol level induced by acute stress stimulation was observed approximately 20 min following the initiation of stimulation. We measured cortisol levels approximately 20 min after the start of suckling, which continued for an average of 16.5 ± 7.0 min, and saliva was collected at an average of 8.0 ± 4.9 min following the cessation of breastfeeding. The sampling time lag before and after breastfeeding was < 35 min. This indicates that the change in cortisol levels before and after breastfeeding was not affected by diurnal change.

### Relationship between Breastfeeding and Perceived Stress

Our study showed that exclusively breastfeeding mothers had lower PSS-10 scores than mothers who practiced mixed feeding. This result supports those of previous studies, which revealed that breastfeeding mothers showed significantly less perceived stress than mothers who bottle fed (Duran, Kaynak, & Karadaş, 2020; Mezzacappa, Guethlein, & Katkin, 2002).

Groër (2005) revealed that breastfeeding mothers have more positive mood and lower perceived stress than those using artificial nutrition. However, breastfeeding mothers had some socioeconomic advantages (age, income, marital status) in Groer’s study. These advantages may translate into stress buffering in real life.

Mezzacappa, Guethlein, Vaz, and Bagiella (2000) stated that breastfeeding status was a significant predictor of PSS score after adjusting for demographic variables. It was reported that 168 breastfeeding mothers had significantly less stress than 65 mothers who had weaned their babies. Systematic reviews showed that breastfeeding is associated with increased parasympathetic nervous system modulation, lower perceived stress levels, and fewer depressive symptoms (Mezzacappa, 2004).

In Foligno et al.’s (2020) study of mothers with preterm infants, a significant negative correlation was observed between high PSS score and reduced rate of breastfeeding during hospitalization. Therefore, breastfeeding seems to enhance both physical and psychological responses to stress.

### Effects of Breastfeeding on Postpartum Depression

We found no association between the feeding method and tendency toward postpartum depression. Previous studies have presented a mixed picture concerning the relationship between postpartum depression and breastfeeding level. For example, Dennis and McQueen (2009) reported that a tendency toward depression during the early postpartum period resulted in increased use of formula, mixed feeding methods, and decreased breastfeeding duration. Nishioka et al. (2011) reported that mothers with tendencies toward postpartum depression changed their infant’s nutrition from breast milk to formula within five months following childbirth, while other studies did not support the relationship between exclusive breastfeeding and lower postpartum depression symptoms at 4–6 weeks (Groër & Davis, 2006) or 4 months (McCarter-Spaulding & Horowitz, 2007).

However, in our study, the finding of a higher PSS-10 score, indicating higher perceived stress, clearly demonstrated a tendency toward postpartum depression. Ahn and Corwin (2015) reported a positive correlation between PSS and EPDS scores 6 months after childbirth. Although our research examined mothers 2–16 weeks following childbirth, our results were similar. Pope, Mazmanian, Bédard, and Sharma (2016) reported that there was no association between the breastfeeding status and a tendency toward postpartum depression but lower household income, lower perceived social support, and higher perceived stress were significantly related to depression status.

### Strengths and Limitations

This study is the first to reveal the relationship between stress reduction and breastfeeding in postpartum Japanese women by measurement of salivary cortisol level and perceived stress. The results indicated acute stress reduction in the hypothalamic-pituitary axis and perceived stress reduction after breastfeeding.

This study had a one group pre-/post-test design of breastfeeding, which may exclude potential bias of setting and individual. However, further control studies on the changes in salivary cortisol levels are required with mothers using artificial nutrition. As we adopted threshold of coefficients of variation <15%, saliva cortisol data of 20 of the 72 participants were excluded. History of depression (antepartum or prepartum) is an important predictor of postpartum depression. This history was not assessed in this study.

This study included Japanese women who live in the regional hub city with uniform life culture, so these findings are not generalizable.

## Conclusion

Salivary cortisol level decreased after breastfeeding, and the longer the suckling time, the lower the salivary cortisol level. Therefore, breastfeeding appears to reduce salivary cortisol level and perceived maternal stress. Although postpartum depression is not related to feeding methods, the tendency toward postpartum depression was significantly associated with increased stress perception.

## Acknowledgments

We express our gratitude to the chief midwife Akiko Aoyama, and her college for assistance with data collections in the Clinic.

## Declaration of Conflicting Interests

The authors declared no potential conflicts of interest concerning the research, authorship, or publication of this article.

## Funding

This study was supported by a Grant-in-Aid for Scientific Research(B) from the Japan Society for the Promotion of Science (JSPS) (Subject No.16H05585 for Mieko SHIMADA).
